# Supplementary material for: Implementation of group interpersonal therapy to treat depression in people living with HIV: A first evaluation of IPT dissemination in Senegal
Source: Glob Ment Health (Camb). 2025 Jun 18;12:e76. doi: 10.1017/gmh.2025.10029 (PMC12322780; doi:10.1017/gmh.2025.10029)
Supplement: Lam et al. supplementary material [file S2054425125100290sup001.docx]

**Supplementary data**

**Table S1: Verbatim illustrating the confirmation of experiences, acceptability, feasibility and benefits of group IPT in *PHASE 2***

| **Themes** | **Verbatim** |
| --- | --- |
| Positive experience/benefits of group IPT | *“From day one, when I saw how people were talking about their problems, I said to myself, wow! (…) I couldn't wait to come back even (...) yes because we're here, we're talking, everyone talks about their problem during the week and that's too good for human beings.*” (PLWH 2)  *“Therapy has allowed us to free ourselves, to talk about our suffering and to find a solution for some of our problems, and even helping a person find a solution to their problem, it makes you happy.”* (PLWH 3)  *“At the beginning I told myself that if the discussions were not useful for me, I was going to stop (LAUGHS), (…) but then I saw its usefulness and I felt good and really comfortable about it, but if I had to receive billions, I would choose therapy because it is to cure me.”* (PLWH 4)  *“Now I have come to my senses, I think less and less about the illness and my existence, and also, I am starting to regain a taste for work (...) I am taking my medication normally, I am starting to eat and drink well again.”* (PLWH 4) |
| Benefits for professionnals | *“Many lessons learned. A lot of experiences. (…) I feel very comfortable now* when *I am around patients. (…) what we can be proud of and make sure of is that anyone who enters the structure and has depression can be easily identified.”* (Facilitator 1) |
| Needs for group IPT | *“He or she (the facilitator) must be able to gain the trust of others and be understanding because it is not easy.”* (PLWH 3)  *“If it's well explained as Mrs. X (facilitator) does, people will understand that there are only advantages in it, they will be motivated to participate and will understand that it's not about exposing one's intimate life but rather about unburdening oneself and clearing one's head.”* (PLWH 5)  *“If it was elsewhere, so as not to lie, I wouldn't be talking (...) here it's discreet and it's around 3:30 p.m., it's time for the descent.”* (PLWH 5)  *“The location is not a problem, but the time is, a little. Because at the time I was summoned, there was a job that I had to finish and I could earn 5000 FCFA there.”* (PLWH 1) |
| Facilitators’ point of view, including benefits beyond depressive symptoms improvment | *“It is feasible if the person who leads has been well prepared, well trained and that there is also the desire to help, the desire to work.”* (Facilitator 1)  "*Well, it's only a form of organization, (...) it's not a burden, it's a mission and it's part of my job.”* (Facilitator 1)  *"It's true that care is of paramount importance, but therapy can also boost a lot compared to the care of PLWH." (*Supervisor 3*)* |

**Table S2: Evolution of depressive symptoms and disability (WHODAS) in function of time***

|  | | | PHQ-9 | | | WHODAS | | |
| --- | --- | --- | --- | --- | --- | --- | --- | --- |
| Predictors | | | Estimates | CI | p | Estimates | CI | p |
| (Intercept) | | | 1.19 | 0.72 – 1.66 | **<0.001** | 12.97 | 12.03 – 13.90 | **<0.001** |
| PHQ-9 scores end of therapy vs baseline | | | 12.18 | 11.60 – 12.75 | **<0.001** |  |  |  |
| PHQ-9 scores end of therapy vs 3-month follow-up | | | 0.02 | -0.58 – 0.63 | 0.936 |  |  |  |
| WHODAS scores end of therapy vs baseline | | |  |  |  | 8.53 | 7.34 – 9.72 | **<0.001** |
| WHODAS scores end of therapy vs 3-month follow-up | | |  |  |  | 0.01 | -1.21 – 1.23 | 0.989 |
| Random Effects | | | | | | | | |
| σ^2^ | | | 3.38 | | | 13.92 | | |
| τ_00_ | | | 1.02 | | | 2.88 | | |
| ICC | | | 0.23 | | | 0.17 | | |
| N | | | 81 | | | 81 | | |
| Observations | | | 228 | | | 226 | | |
| Marginal R^2^ / Conditional R^2^ | | | \| 0.886 / 0.912 \|  \| \| --- \| --- \| | | | 0.497 / 0.583 | | |
| * end of group IPT as the reference | | | | | |  | | |
|  |  |  |  |  |  |  |  |  |

**Table S3: Evolution of depressive symptoms in function of time and Opposite-sex facilitated group*****

| \|  \| PHQ-9 \| \| \| \| --- \| --- \| --- \| --- \| \| Predictors \| Estimates \| CI \| p \| \| (Intercept) \| 1.36 \| 0.78 – 1.95 \| **<0.001** \| \| PHQ-9 scores end of therapy vs baseline \| 12.19 \| 11.46 – 12.91 \| **<0.001** \| \| PHQ-9 scores end of therapy vs 3-month follow-up \| 0.16 \| -0.61 – 0.92 \| 0.688 \| \| Opposite-sex facilitated group \| -0.47 \| -1.45 – 0.52 \| 0.351 \| \| PHQ-9 scores end of therapy vs baseline x Opposite-sex facilitated group \| -0.02 \| -1.23 – 1.20 \| 0.979 \| \| PHQ-9 scores end of therapy vs the end of group IPT x Opposite-sex facilitated group \| -0.30 \| -1.55 – 0.95 \| 0.636 \| \| Random Effects \| \| \| \| σ^2^ \| 3.41 \| \| \| τ_00_ _NUMPAT_ \| 0.98 \| \| \| ICC \| 0.22 \| \| \| N _NUMPAT_ \| 81 \| \| \| Observations \| 228 \| \| \| Marginal R^2^ / Conditional R^2^ \| 0.886 / 0.911 \| \| |  | | |
| --- | --- | --- | --- | --- | --- | --- | --- | --- | --- | --- | --- | --- | --- | --- | --- | --- | --- | --- | --- | --- | --- | --- | --- | --- | --- | --- | --- | --- | --- | --- | --- | --- | --- | --- | --- | --- | --- | --- | --- | --- | --- | --- | --- | --- | --- | --- | --- | --- | --- | --- | --- | --- | --- | --- | --- | --- |
| * end of group IPT as the reference |  |  |  |
